# Explaining chemical clues of metal organic framework-nanozyme nano-/micro-motors in targeted treatment of cancers: benchmarks and challenges

**DOI:** 10.1186/s12951-022-01375-z

**Published:** 2022-03-24

**Authors:** Mojtaba Falahati, Majid Sharifi, Timo L. M. Ten Hagen

**Affiliations:** 1 Laboratory Experimental Oncology, Department of Pathology, Erasmus MC, 3015GD Rotterdam, The Netherlands; 2 Student Research Committee, School of Medicine, Shahroud University of Medical Sciences, Shahroud, Iran; 3 Depatment of Tissue Engineering, School of Medicine, Shahroud University of Medical Sciences, Shahroud, Iran

**Keywords:** Metal organic framework, Motors, Cancer therapy, Propulsion, Biological barriers

## Abstract

Nowadays, nano-/micro-motors are considered as powerful tools in different areas ranging from cleaning all types of contaminants, to development of Targeted drug delivery systems and diagnostic activities. Therefore, the development and application of nano-/micro-motors based on metal–organic frameworks with nanozyme activity (abbreviated as: MOF-NZs) in biomedical activities have received much interest recently. Therefore, after investigating the catalytic properties and applications of MOF-NZs in the treatment of cancer, this study intends to point out their key role in the production of biocompatible nano-/micro-motors. Since reducing the toxicity of MOF-NZ nano-/micro-motors can pave the way for medical activities, this article examines the methods of making biocompatible nanomotors to address the benefits and drawbacks of the required propellants. In the following, an analysis of the amplified directional motion of MOF-NZ nano-/micro-motors under physiological conditions is presented, which can improve the motor behaviors in the propulsion function, conductivity, targeting, drug release, and possible elimination. Meanwhile, by explaining the use of MOF-NZ nano-/micro-motors in the treatment of cancer through the possible synergy of nanomotors with different therapies, it was revealed that MOF-NZ nano-/micro-motors can be effective in the treatment of cancer. Ultimately, by analyzing the potential challenges of MOF-NZ nano-/micro-motors in the treatment of cancers, we hope to encourage researchers to develop MOF-NZs-based nanomotors, in addition to opening up new ideas to address ongoing problems.

**Graphical Abstract:**

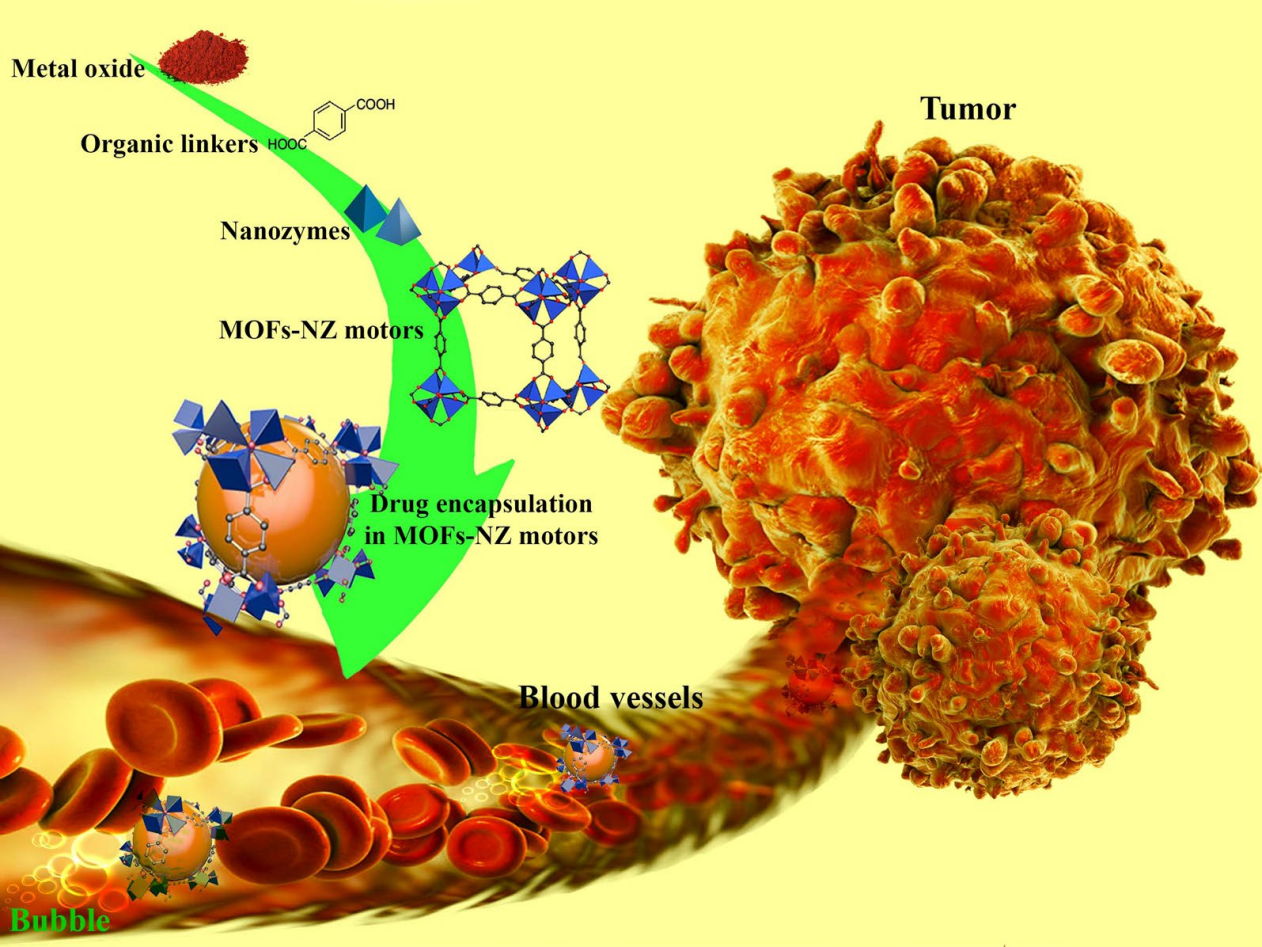

## Introduction

Because invasive activities in diagnosis and treatment of some diseases, such as cancer, cardiovascular disease, bone disease, (chronic) infections, and tissue regeneration, are associated with challenges such as patients refusing treatment and sometimes medical injuries, the use of minimally invasive methods, preferably with high efficiency, have become popular. Although, observations show that invasive activities in diagnosis and treatment of above mentioned diseases depend on several factors such as the skill of the physician or medical staff, facilities and tools used, the nature and location of the disease, diagnostic and therapeutic methods, and patients' behavior during care [1, 2], the use of tools controllable in size and shape, and efficiency of activity, can significantly reduce the level of invasiveness.

Similar to diagnostic settings, therapeutic activities via targeted drug delivery (TDD) to off-target tissue after injection can be considered a form of invasion of tissue or organ [3]. Therefore, using a tool that creates a high concentration of drug or other compounds in the target tissue, without generating pain, can be considered a potential way to increase the well-being of patients. In this regard, Sharifi et al. [4] and Sharifi et al. [5] showed in drug-resistant breast cancer that TDD using nanocarriers not only increased patient well-being by reducing the number of injections and doses, but also provided the development of combination therapies to prevent metastasis, with minimum side effects. Reducing the level of invasion can make medical care easier and recovery time faster. Hence, researchers modeled natural phenomena based on nanotechnology to build molecular engines that, in addition to the ability to detect disease and execute predetermined motor commands, are very effective in development of TDDSs [6–8]. In this regard, molecular machines such as Kinesin and Dynein are observed in the body, which in addition to transferring compounds and biological information to various intracellular regions, cause cell movement and biochemical events [9–11].

Accordingly, significant advances have been reported in the fabrication of nano-/micro-motors with a biological pattern that has been associated with the capabilities of TDDSs [12], system modification [13], and molecular surgery [14]. However, these advances have not yet provided the medical requirements, and so far, the studies reported are merely proving the performance of nano-/micro-motors in laboratory activities. Regardless of design challenges like a propulsion or portable nano-/micro-motors systems, challenges such as size and shape constraints, toxicity due to the presence and retention of NPs, safety responses, environmental complexities in leading tissues, biological barriers (cell wall, proteins, lipids, ions, etc.), propellant fuels of nano-/micro-motors (H_2_O_2_, urea, acids, etc.), hamper the development of nano-/micro-motors for medical application. However, one of the most important challenges is the need for imaging or video simulation in vivo for navigation and activity monitoring of nano-/micro-motors.

Despite these obstacles, it is clear that the use of nano-/micro-motors in the future will be widespread due to safety, higher accuracy even at the cellular level, and unique flexibility in personal medicine [15, 16]. One way to increase the performance of nano-/micro-motors is to use nanozymes (NZs) as metal ions in nano-/micro-motors with organic frameworks. It has already been revealed that NZs with an enzymatic nature are very effective in the treatment of many diseases such as cancers, various superficial and deep infections of the body, cardiovascular diseases, and inflammation [17–20]. Meanwhile, experimental observations have shown the unique capabilities of NZs in imaging, bioassays, and even development of TDDSs [21, 22]. Therefore, the use of NZs in nano-/micro-motors can enhance imaging and vital measurements of nano-/micro-motors by controlling the operating location and accuracy. Although no specific report is available based on our information in this regard, the integration of nano-/micro-motors with NZs can significantly mitigate these challenges.

### Aim of study

In this review, we aim to encourage researchers to integrate and develop extensive use of nano-/micro-motors and MOF-NZs for biomedical activities by focusing on the potential activity of MOF-NZs. In this discussion, after expressing the facts and important questions in the field of nano-/micro-motors based on MOF-NZs, we will give an overview of the catalytic mechanisms, classification, and strategies of improving MOF-NZs activities and their application in medical sectors. Then, we review the design methods of MOF-NZ nano-/micro-motors and their biomedical safety. We will then look at the potential capabilities of MOF-NZ nano-/micro-motors in biomedical practice. Next, the biological barriers against the use of MOF-NZ nano-/micro-motors and the challenges and opportunities of using nano-/micro-motors will be examined. Finally, we draw conclusions from the presented reports and express our view on future developments.

### History of nano-/micro-motors in biomedical applications

Despite the first report of plate motors moving through chemical activity in millimeters scale in 2002 [23], nanomotors, whether metal or organic and hybrid, with the ability to move spontaneously in liquids according to predetermined rules, were first used in 2004 for analyzing activity [24]. However, the idea of using nano-/micro-motors as nanorobots was introduced by Richard Feynman in 1959. Scientific reports show that the use of nano-/micro-motors in TDD started in 2010 [25]. While, nano-/micro-motors biosensing activities where published in 2009–2010 and dual activities of nano-/micro-motors in biological tissues in 2013 [26, 27]. The importance of using macro and nano machines was underscored with the Nobel Prize in 2016 showing that the future of biological and medical application of this technology would instigate significant changes. Therefore, in the last 5 years, there has been a high acceleration in this field. However, the lack of access to comprehensive laboratories and the need for relatively sophisticated technologies are challenging.

### Facts in MOF-NZs nano-/micro-motor

- Because MOF structures have high porosity, responsiveness to pH, and optimal biodegradability under physiological conditions, their use in the generation of bioactive motors in addition to loading the drug in a targeted manner, prevent side effects of the drug carriers [28].
- MOFs with a variety of ligands, metal ions and loading of different metals, have a high potential for catalytic activities to induce motion in the MOF-NZs nano-/micro-motor, based on access to a variety of biofuels [29].
- MOF-NZ nano-/micro-motors as flexible molecular machines can act as switches, shuttles, motors or pumps based on the loading of enzymes and metallic NPs with enzymatic properties. Nonetheless, the coordination of catalytic activities to regulate the velocity and direction of movement to cross biological barriers is still problematic [30].

### Open questions on the use of MOF-NZ nano-/micro-motors

- Which MOF structures are more suitable for the synthesis of MOF-NZ nano-/micro-motors to obtained desired biomedical activities? And by what criteria are these structures evaluated in the different biomedical activities?
- Which chemical fuels and external inducers are more suitable for moving MOF-NZ nano-/micro-motors? And how to create a coordinated, targeted and automatic movement of the motors?
- What are the most important challenges and opportunities for the future use of MOF-NZ nano-/micro-motors in the treatment of cancers?

## MOF-NZs

From the first report on peroxidase-like activity by Fe NPs in 2007 [31], researchers have paid close attention to the use of these metal NPs as NZs in diagnostic and therapeutic applications. Due to the advantages of using metal NZs compared to natural enzymes, such as low cost for synthesis and purification, reusability and high stability, high catalytic sensitivity, and mass production [17, 18, 32, 33], the use of metal NZs in organic framework patterns received more attention in the last decade in the biomedical field as these also show reduced toxicity resulting from increased biocompatibility, easier cleaning, and relatively uniform sizes [34, 35]. MOF-NZs are generally materials with 2D or 3D porosity due to strong and coordinated interaction between organic compounds and metal ions, which in addition to enzymatic properties, can be loaded with drugs and enzymes [36]. Due to the high capacity of these MOFs for loading a variety of compounds and enzymatic activities along with controllable sizes, the implementation of combined heat, electrical and optical treatments and their follow-up through imaging could make MOFs a suitable platform for biomedical activities. In the last decade, several MOF-NZs have been reported to have similar and occasionally enhanced catalytic activity compared to bare enzymes [37, 38]. For example, recently Wu et al. [39] designed a MOF-NZ with the chemical structure MIL-47(V)-NH_2_, which mimics glutathione peroxidase activity by reducing inflammatory responses in ear injuries and colitis. In this regard, the use of MOF-NZ nano-/micro-motors for therapeutic application, especially in the field of cancer, has received much attention in the last two decades, despite the slower progress compared to other nanomotors [40]. While MOF-NZ nano-/micro-motors not only allow the execution of extracorporeal commands based on magnetic or electric fields, ultrasound, light or a combination of these based on the presence of metal compounds, also targeting of therapeutic activities is possible because of the favorable interaction with chemicals [41]. However, not all MOF-NZs can be recognized as nanomotors due to the lack of regular and controllable motion in natural systems. Therefore, in addition to focusing on the above two factors, the biocompatibility features, therapeutic loading capacity, delivery efficiency, tracking, and biodegradation of nanomotor activity in therapeutic activities has complicated the use of MOF-NZ nano-/micro-motors. For example, it has recently been shown that biocompatible (NaYF4@NaYb0.92F4:Er0.08@NaYF4)Zr/Fe porphyrin nanomotors decorated with AuNZ in addition to GOx activities to detect glucose, are very effective in eliminating cancer cells by producing single oxygen and inducing starvation [42]. Multi-purpose activities in this category of MOF-NZ nano-/micro-motors can provide the way for more accurate and effective therapeutic activities.

### Classification of MOF-NZs

In general, based on manufacturing procedures, MOF-NZs are divided into three sections, which include pristine MOFs, MOF-based nanocomposites and MOF-based derivatives. MOF-NZs produced according to the pristine MOFs, which include metal cluster nodes and organic ligands, not only provide the enzymatic activity required for biomedical application such as cancer therapy, but also enhance the possibility of multi-enzyme activity based on the types of metal ions used [43]. Nonetheless, the results of published reports indicate that the unexpected digestion and limited solubility (under physiological conditions) of NZs generated based on pristine MOFs will be a serious challenge in therapeutic and diagnostic use [44]. Although the nanocomposite procedure for the production of MOF-NZs faces the same challenges, the use of this technique has received much attention due to the increase in the loading of enzymes and the simple surface modification of the MOFs [45]. However, the reports presented in this procedure show that despite the much higher transfer of enzymes and metallic dots loaded on MOF-NZs, their instability and poor performance compared to natural enzymes are considered as a drawback in physiological conditions [46]. Therefore, researchers have shown great interest in MOF-based derivatives, which include nano porous carbon, metal/carbon, metal oxide/carbon, metal/metal oxide/carbon. MOF-based derivatives, due to wider surface, higher stability, significant reduction of agglomeration, more ordered porous structure, higher active site, and due to loading of different materials, simple adjustment in amount of porosity, and the controllable sizes of the cavities [47], effective properties in the catalytic activity and loading of the drugs or enzymes, compared to others MOFs, are very important.

### Catalytic mechanisms

Although the mechanism of MOF-NZs performance, like enzymes, can be divided into two families: (1) oxidoreductases including oxidase, peroxidase, SOD, CAT and nitrate reductases, and (2) hydrolases including nuclease, phosphatase, protease, esterase, the most common mechanisms studied in the therapeutic activities are oxidase, peroxidase, CAT, and SOD as shown in Table 1.

**Table 1:** Common enzymatic mechanisms in MOF-NZs for therapeutic and diagnostic activities

Function	Formulation	Application	Refs.
Oxidase	TPZ-GOx-ZIF-8@eM	Tumor therapy by Starvation/CHT in CT26 tumor xenografts model	[48]
	Mem@GOx@ZIF-8@DOX	Tumor therapy by Starvation/CHT in 4T1 tumor xenografts model	[49]
	PCN@Pt@PCN-Au-FA	Tumor therapy by Starvation/PDT in 4T1 tumor xenografts model	[50]
	GOx@ZIF@MPN	Tumor therapy by Starvation/CDT in 4T1 tumor xenografts model	[51]
	Co‐Fc@GOx	Tumor therapy by Fenton reaction/CDT in 4T1 tumor xenografts model	[52]
	GOx-Hb@ZIF-8	Tumor therapy by Fenton reaction/starvation therapy in MCF-7 and HeLa cancerous cells model	[53]
	AuNPs-Fe@GOx	Tumor therapy by PTT in microenvironment tumor model	[54]
Peroxidase	DOX@MIL-100@HA	Tumor therapy by CDT/CHT in MCF-7 tumor xenografts model	[55]
	IL@MIL-101(Fe)@ BSA-AuNCs	Tumor therapy by CDT/ PDT therapy in H22 liver tumor xenografts model	[56]
	DOX@PCN@-MnO2@PAH	Tumor therapy by glutathione-depletion /CHT/PDT in 4T1 tumor xenografts model	[57]
	BPQD/HKUST-1@MIL-100 (Fe)-GSNO	Tumor therapy by CDT/gas therapy/PTT in human gastric tumor xenografts model	[58]
	UsAuNPs/MOFs	Antibacterial therapy in antibacterial properties against both Gram‐negative and Gram‐positive bacteria model	[59]
CAT	BQ-MIL@CAT-MIL	Tumor therapy by O_2_-evolving/PDT/PTT in human cervical cancer tumor xenografts model	[60]
	mZIF-8@-CAT@DOX	Tumor therapy by O_2_-evolving/CHT/IMT in mouse melanoma tumor xenografts model	[61]
	BM@NCP(DSP)-PEG	Tumor therapy by O_2_-evolving/radiotherapy/IMT in 4T1 tumor xenografts model	[62]
	MOF-MBDHA@PLA@PEG	Tumor therapy by O_2_-evolving/PDT/CHT in mouse cervical tumor xenografts model	[63]
	MIL-100/GOx@HA-PDA	Tumor therapy by CDT/PTT/starvation in 4T1 tumor xenografts model	[64]
SOD	PVP@CeNPs@MIL-100	Alzheimer therapy by CDT in AD mouse model	[65]
	Aptinib@PCN@MnO2@Tm	Tumor therapy by GSH-depletion/PDT/antiangiogenesis in 4T1 tumor xenografts model	[66]
	Pt@PCN222-Mn	Anti-inflammatory by CDT in IBD of mouse model	[67]
	Cu-TCPP-MOFs/nanodosts	Anti-inflammatory by hemodynamic therapy in an endotoxemia model in vivo	[68]
	PCN-224(Cu)GOx@MnO2	Tumor therapy by GSH-depletion/CHT/anti-angiogenesis in U14 tumor xenografts model	[69]
	NH_2_- MIL-88B (Fe)	Tumor therapy by GSH-depletion/nano therapy in A375 and HeLa tumor xenografts model	[70]

The enzymatic performance of MOF-NZs is accompanied by slight changes in activity as compared to bare NZs due to the presence of organic compounds such as 1,4-benzene-dicarboxylic acid [71], Fumaric acid [72], TCPP [73], 2-methylimidazol [74], 2-amino-terephthalic acid [75], 2,2′-Dithiosalicylic acid [76], Nucleotide [77], and 4,4′-Bipyridine [78] in the structure. Metal NPs provide catalytic activity according to the metal-based redox couple. Whereas, organic compounds exhibit catalytic activity, like natural enzymes, based on the acceptance of electrons from one substrate and transfer to another substrate. Overall, this classification is only for an easier understanding of the activity of NZs in the MOFs structure for biomedical activities.

**Fig. 1:**
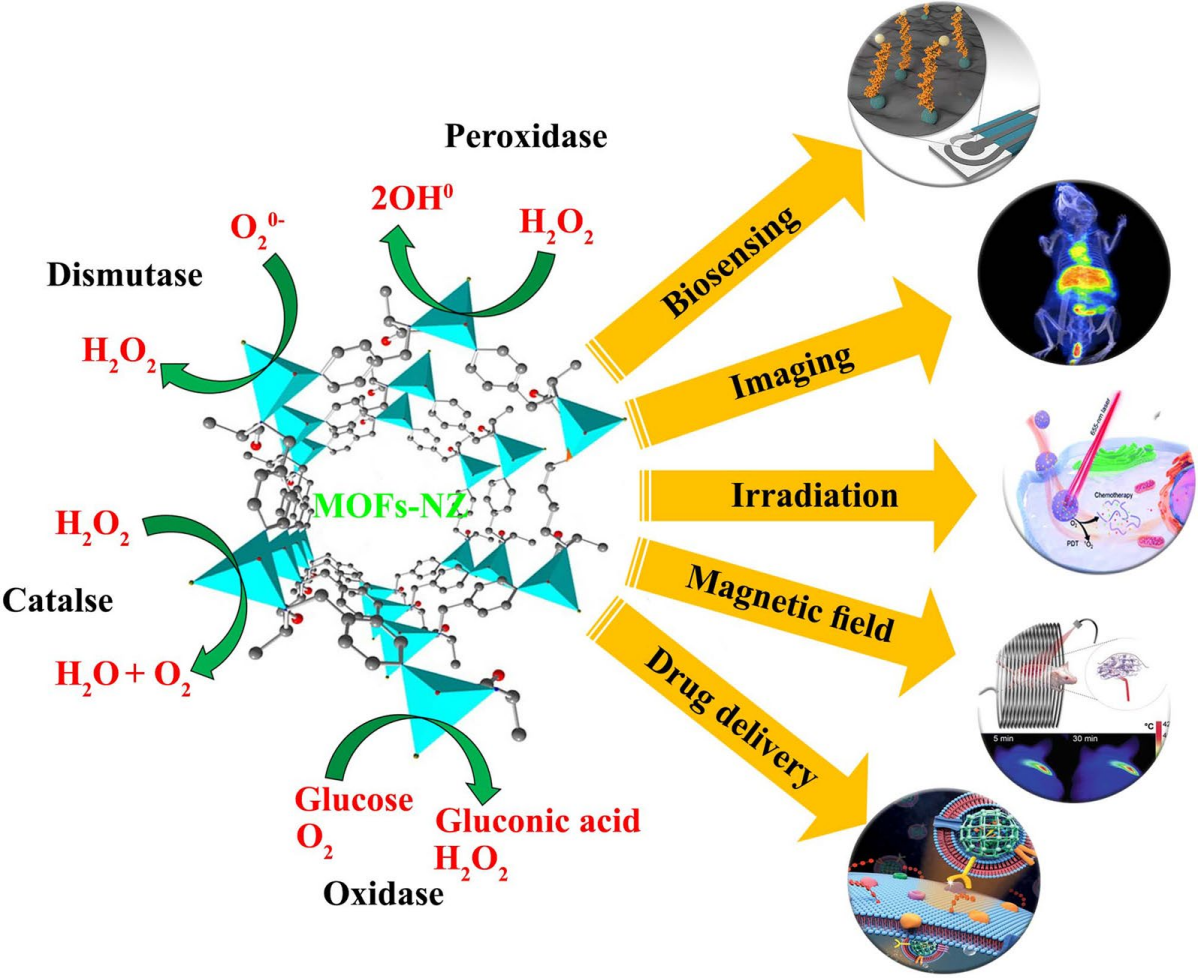
Mechanism of catalytic activity of MOF-NZs and their biomedical application

In the mechanism of oxidase (Fig. 1), which was first discovered by Rossi et al. [79] in 2004, substrates such as cholesterol, sugar, uric acid, and others are oxidized by molecular oxygen. In this mechanism, the metal NZs produce substances such as H_2_O_2_, water, and sometimes radio-active-superoxide by transferring electrons to molecular oxygen [80]. Due to the simplicity of the mechanism in the oxidase mechanism, the use of this method for enzymatic activities in the MOFs structures seems to be very desirable based on the publications by Yuan et al. [81] and Li et al. [38] with Fe and CuO NZs, respectively. It seems that the use of MOF-NZ nano-/micro-motors with oxidase as functional mechanism can be very desirable for the movement of nanomotors in therapeutic activities due to the abundance of glucose and urea propellants. On the other hand, the observed glucose concentration gradient in tumor tissues can be considered as one of the important factors for MOF-NZ nano-/micro-motors targeting [82]. Moreover, the peroxidase mechanism (Fig. 1), which is one of the vital reactions of the Fenton class, catalyzes the substrate with H_2_O_2,_ or other compounds containing H_2_O_2_, as electron acceptors and digests the peroxide compounds [83]. Glutathione peroxidase and halo-peroxidase are important enzymatic reactions in the peroxidase family that play an important role in biosensing activities and immune system enhancement [84, 85]. The most common NZs used in MOFs structures for peroxidase activities are Fe, Pt and Mn NPs [86], which were first used by Gao et al. [31]. Peroxidase activity of Fe, Pt, and Mn NZs doped nanomotors, with H_2_O_2_-based chemical propulsion, are interesting pathways in research activities due to the enhanced performance by optical inductors [87]. In this regard, the first Au/Pt nanomotors with a length of 2 µm and a diameter of 370 nm were designed with H_2_O_2_ propulsion, for which directionality was still a challenge [88]. However, Laocharoensuk et al. [89] by replacing carbon nanotubes instead of Au, were able to increase the speed of the H_2_O_2_ fueled nanomotors (50 − 60 µm/s), in addition to improving the directionality. Therefore, the use of peroxidase mechanism in the motion of MOF-NZ nano-/micro-motors can act with higher persistence due to the accumulation of H_2_O_2_ in tumor tissue, especially in solid tumors, regardless of the challenge of H_2_O_2_ available in the healthy environment.

Mechanism of SOD (Fig. 1) is to actively remove produced free radicals by convertion to peroxide and water, and as a result the inflammation caused by oxidative stress is significantly reduced. In this regard, it can be seen that CuO and ceria are the most important NZs used in the MOFs structures based on this mechanism [90]. Importantly, the presence of CuO NPs in the body for a longer period of time has shown very low toxicity [91]. The last very common reaction in NZs is the CAT mechanism (Fig. 1), which is manifested by the degradation of peroxide to oxygen and water. This mechanism plays a vital role in reducing cellular oxidative stress and was first used in the structure of MOFs by Shieh et al. [92]. Today, the enzymatic reactions of the hydrolase family are highly regarded. In this regard, Chen et al. [93] were able to effectively perform esterase activities by designing Zr and Zn based NZs in MOFs structures.

In this section, we briefly introduce the catalytic mechanisms of NZs that can be loaded onto the MOFs structures. To better understand the catalytic activity of NZs, we refer to reports published by Huang et al. [83]. In general, it has been described that the activity of NZs significantly depends on NPs size and shape, NPs composition, surface modification with ions, coatings or loading with additional compounds, and specifically the environment in which NZs function.

### Strategies of improving MOF-NZs activity

Since the activity of MOF-NZs, like enzymes, is based on substrate adsorption, diffusion, catalytic activity and output, strengthening of each of these items, can significantly improve the activity of MOF-NZs. In this regard, several reports show that reducing the size, improving surface charge, surface modification and chemical structure of MOF-NZs effectively improve the enzymatic activity [36, 94, 95]. For example, Cheng et al. [96] displayed that the catalytic activity of 2D Zn-tetrakis(4-carboxyphenyl)porphyrin(Fe) not only increased the enzymatic kinetic rate compared to 3D, but also improved the reaction rate by twofold. They found that increasing the surface-to-volume ratio improves catalytic activity by enhancing the adsorption process of the substrate, which is the first step in the reaction. They found that diagnostic sensors based on 2D Zn-tetrakis(4-carboxyphenyl)porphyrin(Fe) modified with AG73 peptides (with high specificity for heparin) were able to detect the process of heparin change in the blood of mice with a limit of detection of 15 ng/mL and a linear range of 0.1–10 µg/mL [96]. On the other hand, Wang et al. [97] revealed that reducing the size of Zr porphyrin MOFs in addition to improving stability, strengthens their catalytic process by increasing the MOFs solubility and adsorption process to the substrate. They also showed that enhancing the surface charge of Zr porphyrin MOFs through the solvothermal technique could increase the substrate adsorption process which enhanced the catalytic activity. Based on the properties of GOx of designed MOF-NZs, it was possible to measure glucose in real samples of human serum effectively and with high accuracy, to regulate diabetic problems, especially in complex biomatrix. In this line, Liu et al. [98] by surface modification of Fe-porphyrin-based porous organic polymers (PPOPs) through sulfonic acids were able to improve the catalytic activity by increasing the solubility of Fe-PPOPs-SO_3_H. This was achieved by enhancing the aromatic electrophilic activity and by increasing the affinity of the substrate to MOFs by amplifying the negative charge of Fe-PPOPs-SO_3_H. The MOF-NZs were produced as a colorimetric sensor, in addition to detecting H_2_O_2_ with a detection limit of 26.70 μM (a linear range of 50–1800 μM), were able to detect glucose very quickly with a detection limit of 16.38 μM (a linear range of 200–1500 μM) [98]. In addition to NPs size and surface modification, Dang and Zhao [99], Hu et al. [100] and Wang et al. [101] confirmed that catalytic activity is effectively increased by altering the chemical structure of the MOFs through the integration of various metal NPs such as Fe, Pt, Zr, Co, Mn and Ni. They showed that the use of a bimetallic MOF structure instead of a mono-metallic increases the catalytic activity due to the amplification of the active sites and the redox electron transfer between the two metal sites. It seems that the integration of these methods such as changing chemical structure, together with size and surface modification, can effectively increase the catalytic activity of MOF-NZs.

### Application

#### Biosensing and bioimaging

The range of diagnostic activities including enzymatic substrates such as H_2_O_2_, biomolecules such as sugar, inorganic ions, genomic compounds, and reducing species such as bio-thiols in MOF-NZs by calorimetric, electrochemical, fluorescent, SERS, and chemiluminescence methods, performs simple diagnosis without the need for multi-step methods in bio-matrixes efficiently and accurately (Table 2). Although bioassay activities by MOF-NZs are based on in-vitro activities, the use of MOF-NZs in bioimaging like MRI, CT, US and PET is also conceivable (Table 2). For instance, using MIL-100(Fe,Mn)@PEG-CO-DOX (50 nm), Yao et al. [102] were able to amplify T-2 in MRI imaging, which not only resulted in higher contrast images of colon cancer after intratumoral administration, but also MOFs could when used in combination with photothermal therapy (PTT), release doxorubicin (DOX) controlled in the tumor. In this regard, Yang et al. [103] recently showed that DOX@MoS_2_-PMA nanoplatform with photoacoustic imaging and MRI capabilities can produce better imaging along with controlled drug release and PTT activities compared to free drug and non-mesoporous naked NPs.

**Table 2:** Biosensing and bioimaging applications of MOF-NZs

Materials	Detection	LOD	Range	Ref
Colorimetric				
Fe-MIL-88NH_2_	Glucose	0.48 μM	2 -300 μM	[104]
Fe-MIL-88A	Thrombin	< 10 nM	10–80 nM	[105]
Fe-MIL-88FA	Acid Ascorbic	15 μM	30–1030 μM	[106]
RIgG@Cu-MOF	mIgG	0.34 ng/mL	0–100 ng/mL	[107]
Ni-hemin MOFs	Breast cancer	10 cells/mL	50–10^5^ cells/mL	[108]
Zr-MOF-ssDNA-AuNP	Infertility	90%	unknown	[109]
Electrochemical				
Au@Pt/MIL-53-HRP/hemin/G-quadruplex DNAzyme	COVID-19	8.33 pg/mL	0.025–50 ng/mL	[110]
Fe_3_O_4_@UiO-66/Au@PtNP	Cardiac troponin I	5.7 pg/mL	0.01–100 ng/mL	[111]
PdNPs@Fe-MIL-88NH_2_	miR-122 (liver injury)	0.003 fM	0.01 fM-10 pM	[73]
MB@DNA/UiO-66-NH_2_	Carcino-embryonic antigen	16 fg/mL	50 fg/mL-10 ng/mL	[112]
Zr-UiO-66-2NH_2_/ PO_4_-Apt	Breast cancer	31 cell/mL	10^2^–10^4^ cell/mL	[113]
cDNA/CoNi-MOFs	miRNA-126	0.14 fM	–	[100]
Fluorescent				
Cu-MOF-199	Thiamine	1 μM	4–700 μM	[114]
MIL-53(Fe)	Glucose	< 7.54 nM	0.5–24 μM	[115]
MIL-53(Fe)	Alkaline phosphatase	0.7 U L	2–80 U/L	[116]
ssDNA/ZIF-8/Ag nano-clusters	miRNA	Ultrasensitive	0.175–500 pM	[117]
NH2-Cu-MOF	Hypoxanthine	3.93 μM	10–2000 μM	[118]
CuBDC nano-structure	Pyrophosphate	0.6 mU·mL	1–50 mU/mL	[119]
SERS				
MOFs@Au tetrapode@TB/Ab	Acute pulmonary embolism	0.75 fg/mL	1 fg/mL-1 ng/mL	[120]
MOF@TB@cDNA Y-junction	ATP	0.4 nM	1–200 nM	[121]
AgNPs/MIL-101(Fe)/ABTS	Dopamine	0.32 pM	1.05 pM- 210 nM	[122]
Au@Hexaphosphate@MIL-101	Methenamine	5.0 × 10^–10^ M	3.16 × 10^–6^- 1.0 × 10^–8^ M	[123]
Cu_2_O@SiO_2_@ZIF-8@Ag	Polyaromatic structures	5.7 × 10^–12^ mol/L	Unknown	[124]
AuNPs@MIL-101@Lactate Oxidase	Lactate	5 μM	100–200 μM	[125]
Chemiluminescence				
MIL-100 (Fe)/Apt	α-fetoprotein	7.7 × 10^−11^ g/L	1 × 10^−10^–3 × 10^−5^ g/L	[126]
Hemin@HKUST-1	Glucose	50 μM	75–1000 μM	[127]
NH_2_–MIL–101(Al) MOFs	Fluoride ion	0.05 μmol/L	0.5–80 μmol/L	[128]
Ru@MOFs/CNT-Ferrocene	m^6^A-RNA	0.0003 nM	0.001–10 nM	[129]
Tb@Zn–MOFs	Alkaline phosphatase	0.05 mU/mL	0.1–70 mU/mL	[130]
Au&Pt@UiO-66	Protein kinase A	0.009 U/mL	0.01–0.25 U/mL	[131]
Bioimaging				
ICG-CpG@ MIL101-NH_2_	Photoacoustic imaging and MRI methods	Breast cancer (4T1)		[132]
Gd-BTC-MOF@SiO_2_-^10^B	MRI method	Solid tumors		[133]
^177^Lutetium-PCN-PEG	CT method	Breast cancer		[134]
FeN200@GOx@M	US method	Ovarian cancer		[135]
^64^Cu-DOX-AZIF-8	PET and CT methods	Breast cancer (4T1)		[136]
MIL-101(Fe)@sorafenib	MRI method	Hepatocellular carcinoma		[137]
DOX@GNRs-MSNs-HA	MRI, Photoacoustic imaging and CT methods	Breast cancer (4T1/MCF-7)		[138]

#### Cancer therapy

Although the administration of nanocarriers varies depending on the type of treatment, like a TDD, photo-therapy, thermal-therapy, and radio-therapy, the mechanism of the cancer treatment process by MOF-NZs generally follows five general modes, including control of hypoxia in tumors based on catalytic activity, an increase in toxic agents such as H_2_O_2_ induced by Fenton reactions, tumor starvation by lowering glucose levels through GOx activity, a decrease in GSH to increase intracellular ROS, and reinforcement of catalytic cascades via synergistic effects. In summary, the five methods are reported in Table 3.

**Table 3:** A summary of the MOF-NZs used in the treatment of cancer

Materials	Tumor model	Treatment technique	Inhibition	Ref
O_2_-evolving				
UiO-66-NH_2_/PB-DOX	Breast cancer	PTT/CHT	80% <	[139]
Zr_6_C_72_H_45_N_6_O_12_-Mn_1.5_	Liver cancer	SDT/IMT	89% <	[140]
TPP-DNB@ZIF-8	Breast cancer	PDT	85% <	[141]
TPZ@porphyrinic MOFs	Colon cancer	PDT/CHT/IMT	87% <	[142]
AuNPs-Fe@GOx	Lung cancer	PTT	80% <	[54]
Toxic agents				
HA@MIL-100(Fe)/D-Arginine	Osteosarcoma	RT	90% <	[143]
Cu-MOFs/Ce6	Breast cancer	CDT/SDT	75% <	[144]
PCN-224-Pt	Liver cancer	PDT	78% <	[145]
Zr-Fc MOFs	Breast cancer	PTT/CDP	90% <	[146]
Starvation				
Banoxantrone/GOx@ZIF-8@Cell membrane	Liver cancer	CHT	85% <	[147]
Carbone-oxide framework@GOx-CAT	Breast cancer	PDT	80% <	[148]
MnO_2_ nanosheets-GOx	Melanoma cancer	PDT	90% <	[149]
GOx@Pd@ZIF-8	Lung cancer	CDT	50% <	[150]
Fe-hemoporfirin frameworks–GOx/CAT	Breast cancer	SDT	75% <	[151]
GSH depletion				
MnFe_2_O_4_@Zr-TCPP-PEG	Breast cancer	PDT	75% <	[152]
Fe·Cu-SS-PEG@DOX MOFs	Breast cancer	CDT/PTT/CHT	70% <	[153]
PCN-224(Cu)-GOD@MnO_2_	Cervical cancer	CDT	90% <	[154]
mFe(SS)/GSH MOFs	Breast cancer	CHT/IMT	75% <	[155]
Cu‐Pd@MIL‐101	Solid tumor	CDT	70% <	[156]
Catalytic cascades enhanced				
P@Pt@P–Au–FA	Breast cancer	PDT: GOx + CAT	75% <	[50]
GOx@ZIF@Metal polyphenol	Breast cancer	CDT: GOx + CAT + Fenton	85% <	[157]
Mn-Zr(MOFs) nano-clusters	Liver cancer	MDT: GOx + CAT	85% <	[158]
siRNA/Zr-Fe–P MOFs	Breast cancer	PTT/PDT: GOx + CAT	80% <	[159]

## MOF-NZ nano-/micro-motors

Despite the production of nano-/micro-motors combined with biomaterials such as proteins that move through ATP consumption, the production of micro-/nano-motors based on enzymatic activity was started with a slight delay in 2008 by Sundararajan et al. [160]. These motors with the enzymatic activity of Au-Pt on H_2_O_2_ provided the necessary force to move the nano-/micro-motors using oxygen bubbles. Accordingly, the use of catalytic activities of other metal NPs in nano-/micro-motors, such as Fe, Ir, Al, Zn, and Pd, on biological compounds was also considered [161]. However, the use of nano-/micro-motors in therapeutic setting faces many limitations due to the challenges of biocompatibility and biodegradability, lack of complete control over nano-/micro-motors in physiological fluids, and the size of nano-/micro-motors along with the fuels used to drive nano-/micro-motors. Nevertheless, the use of NZ-based nano-/micro-motors is much more reliable in therapeutic and diagnostic activities compared to other nano-/micro-motors due to higher biocompatibility and biodegradability, frequent use, higher specificity and selectivity in physiological fluids, the possibility of simple activation or deactivation, and the use of common non-toxic fuels in the body based on the enzymatic properties of CAT, lipase, urease, GOx, and ATPase.

### Critical rules in the design of MOF-NZ nano-/micro-motors in vivo

Although the design of enzymatic nano-/micro-motors by mimicking the bio-engines present in the body is complex and costly, the ability to perform highly accurate medical activities has provided a promising platform for the treatment of incurable patients in the molecular and intracellular fields as well as non-invasive surgeries. Because controlling the behavior of nano-/micro-motors, such as Brownian and progressive motion, which in addition to fuel depends on the geometry and size of the nano-/micro-motors and even the components, precise design is crucial. Asymmetric geometry or improper accumulation of catalytic components on nano-/micro-motors can cause asymmetric motion of the motors. For example, Patiño et al. [162] showed that changing the distribution of the urease loaded on theta SiO_2_-coated polystyrene (PS@SiO_2_) motors changes the linear motion and even the speed of motors compared to the controlled distribution of the enzyme on the motor surface. Their results revealed that the increase in velocity and force of the motor depends nonlinearly on the increase in the concentration of the urease coating [162]. Also, based on the hypothesis of greater and easier penetration of 20 to 250 nm particles into the cell, it was found that changing the micrometer (1 µm) size to nanometers (150 nm) by molding with PEG during production increased the amount of penetration of small polymersomes into stomatocytes in a vasculature model [163]. On the other hand, the removal of nano-/micro-motors after pre-designed activities and their biocompatibility during therapeutic and diagnostic activities are very important [164]. Therefore, the mentioned challenges including shape/size, biocompatibility and biodegradability, do not allow the use of all the common techniques in MOF-NZ nano-/micro-motors design.

#### Fabrication

Since the synthesis of MOF-NZs with uniform cavities, homogeneous particle size distribution, and stability is challenging enough, the synthesis of nano-/micro-based motors seems very ambitious and sometimes impossible. Despite various methods of MOF-NZs synthesis such as solvothermal, electrochemical, microwave, sonochemical, atomic layer deposition, ionothermal, spray dryer, sol gel, ultrasound radiation, modulating agents, grinding, and microfluidic techniques [165], MOF-NZs synthesis based on wet chemical approach with the premise of controlling nucleation and directional growth of crystals in chemical solutions is very simple and relatively common. In this regard, the use of wet chemical approach in the production of MOF-NZ nano-/micro-motors seems simpler, cheaper and more feasible. Therefore, despite the different techniques of synthesis of nano-/micro-motors in the form of two general methods of assembly and non-assembly, the most common technique used for the synthesis of MOF-NZ nano-/micro-motors in biomedicine is the assembly technique based on wet chemical approach. This technique not only provides an easy and effective process for loading drug or enzymatic compounds, but also makes it possible to easily regulate the morphology and size of MOF-NZ nano-/micro-motors. In this regard, based on the report of Wilson et al. [166] polymeric compounds such as amphiphilic polymers of PEG-b-polystyrene can be used, which have a high ability to control deformation. In this process, catalytic NPs such as Pt can optimally enter the external cavities created in polymers and form the nanomotor structure with a 400 nm dimension. After depositing the NPs in the cavities and removing the polymer pattern, spherical or tubular nanomotors are extracted. In addition to polymeric compounds, the use of silica and surfactant precursors based on the "sol–gel" process in the production of nanomotors has been reported to be desirable. In this regard, Ma et al. [167] were able to provide different sizes of semi-coated nanomotors with dimensions of < 100 nm using mesoporous silica NPs and deposition of a 2 nm layer of Pt on one side. The use of hollow structures by silica precursors promises high loading capacity of drug compounds and so on. In this method, next to spherical motors, it was possible to make easy Janus and tubular motors. In this line, Tan et al. [168] after producing 200 to 500 μm crystals of ZIF-8 and ZIF-67 from Zn(NO_3_)0.6H_2_O, 2-methylimidazole and poly[vinylpyrrolidone], were able to make Janus ZIF-8/ZIF-67 micromotors based on the molding of poly[methyl methacrylate], which moves by catalytic decomposition of H_2_O_2_ by generating bubbles. The speed of the MOFs micromotors provided was 1 mm/s. Regulating the deposition of NZs on tubular motors makes it easy to move forward. Another route for assembling micromotors was introduced by Ikezoe et al. [169], which is based on self-assembled diphenylalanine in the [Cu-1,4benzenedicarboxylate-triethylenediamine]_n_ structure as a MOF. Since the energy source in this MOF-NZ micromotors is based on the free energy change of diphenylalanine release, it is very similar to biological motors, which in general has a mechanical movement against the slope of the environment due to energy storage through the release of hydrophobic diphenylalanine and Marangoni movement over time. In this regard, the first MOF-NZ nanomotors were introduced by Dong et al. [170] based on the deposition of Au, Fe and Pt NPs on poly-caprolactone polymer single crystals (PSC). By designing PSC-Au-Pt-Fe_3_O_4_ nanomotors with dimensions of 5–10 nm based on Pt activity, they were able to increase the nanomotor speed to 30 µm/second. Although various methods can be seen in the assembly process, such as the integration of NPs into biological cells and even biological compounds such as DNA [171], the use of polymeric and MOFs methods in the design and production of biological nano-/micro-motors seems much more desirable than other methods due to the simpler process and easier maintenance.

#### Biocompatibility

One of the most important challenges in using MOF-NZ nano-/micro-motors is the biocompatibility of the elements used, which has forced researchers to use biocompatible or biodegradable compounds. The best way to study the biocompatibility of nano-/micro-motors is to track the response of host tissues to nano-/micro-motors. To reduce the immune response, researchers recommend the use of biodegradable metal NPs (Mg, Zn, Fe, etc.), biodegradable organic frameworks (amino acids, peptides, organic acids, etc.), and biocompatible polymer coating (PEG, PCL, PVA, etc.) [172]. For instance, Wu et al. [173] and Wu et al. [174] designed nano/micro-motors based on a layer-by-layer technique with Fe_3_O_4_, Pt and Au NPs as catalysts in the framework of chitosan/sodium alginate and albumin/poly-l-lysine to deliver DOX to breast cancer cells by combining bio-catalytic bubble propulsion and magnetic conduction at a speed of ~ 22 and 68 μm/s, respectively. Compared to synthetic nanomotors, which are previously composed of non-degradable metal NPs, these nanomotors have high biocompatibility and good biodegradability due to the polymers and Fe NPs used. Also, it was shown that MOF-NZ nanomotors (HKUST-1: Cu_3_(1,3,5-benzene tricarboxylate)_2_) containing hydrophobic diphenylalanine (DPA) peptides produced by self-assembly with pore size of 0.75 nm in addition to high stability in water have good biocompatibility for medical activities [175]. The engine propulsion mechanism is based on hydrophobic amino acid release and a high surface tension gradient. In addition, Gao et al. [176] designed CAT-succinylated β-lactoglobulin@ZIF-L (CAT-β@ZIF-L) micro motors whose motion was regulated by the acidity of the environment based on the response of CAT and succinylated β-lactoglobulin to acidity. At pH < 6 succinylated β-lactoglobulin allows the permeability of fuel (H_2_O_2_) into the micromotors for CAT activity in order to move the micromotors. At neutral pH between 6.5 and 7.2, gel-induced succinylated β-lactoglobulin reduces enzymatic activity by limiting fuel availability. Despite the control of direction and speed of the MOF-NZ micromotors by the slope of environmental acidity and low toxicity, the challenge of the level of acidity required in body tissues which can be considered as NZs, the MOF-NZs nano-/micro-motor error in detecting the target tissue due to various acidity in different organs, as well as the direction or strength of nano-/micro-motor motion in tissues without limit of acidity threshold, remains unanswered.

### Propulsion of MOF-NZ nano-/micro-motors

To achieve progressive movement in nano-/micro-motors, Brownian and low Reynolds motions should be overcome. For this purpose, two general strategies are used, which include chemical propulsion and external stimulation. The chemical propulsion of motors is based on the asymmetric chemical reaction of the surface with the fuels described in Table 4, and the creation of a driving force based on the exit of bubbles in the opposite direction of motion. This type of propulsion is challenged due to the biological limitations and short life of metal NPs as a catalytic source along with production complexities. The biological limitations of propellants are based on the by-products of fuel decomposition (Table 4) and the creation of additional inflammation in tissues with unpredictable reactions. For example, MOF-NZ nanomotors based on ZIF-8 and Pt require a concentration of more than 25% H_2_O_2_ as a propellant fuel to move, which is not possible in a biological setting due to the intrinsic toxicity [177]. However, Wang et al. [178] and Liu et al. [179], by designing a MOF-NZ nanomotors based on ZIF-67 and ZIF-8, and Fe_3_O_4_ instead of Pt, were able to significantly reduce the toxicity of the nanomotors by reducing the required level of fuel (H_2_O_2_) by up to 10% by merging with a magnetic field. Therefore, the integration of different propulsion procedures such as chemical fuels (H_2_O_2_, urea, and acids) and external fields (irradiation, ultrasound and magnetic fields) can be effective in MOF-NZ nano-/micro-motors biocompatibility. Hence, the use of external stimuli including magnetic and light radiation are considered to overcome the challenges ahead.

**Table 4:** Fuels types of MOF-NZs micro-/nano-motors

H_2_O_2_	Properties	H_2_O_2_ is available in all aerobic metabolism, and it is generated via an extracellular and intracellular proceeding. The mitochondrial electron transport chain, the arachidonic acid metabolizing lipoxygenase and cyclooxygenase, the cytochrome P450s, xanthine oxidase, NAD(P)H oxidases, uncoupled nitric oxide synthase and peroxidases can be potential sources of H_2_O_2_ enzymatic. Tumor cells generated more H_2_O_2_ than normal cells
	Limitation	H_2_O_2_ is harmful to cells when it reaches a certain concentration of 50 µM < , resulting in the oxidation of DNA, lipids and proteins
	Mechanism	H_2_O_2_ is decomposed into water and oxygen bubbles, that oxygen bubbles provide the force to motors as a bubble propulsion
Water	Properties	Water is a liquid available in various tissues and biocompatible that can be highly regarded as a local fuel for nano-/micro-motors. The hydrogen bubbles resulting from the reaction between the active metal NPs such as Mg, Pt, Al, Ti, Ga, and water are responsible for propelling
	Limitation	Accumulation of H_2_ in the tissue increases the possibility of acidification in the presence of CO_2_. On the other hand, despite the positive value of H_2_ accumulation in tissues to reduce free radicals and reduce apoptosis, in cancer tissues this feature will be considered as a negative factor
	Mechanism	Based on the chemical reaction between nano metals and water
Urea	Properties	Urea, which is generally produced by amino acid catabolism, is a source of excreted nitrogen to reduce nitrogen toxicity in the body. Urea is produced in the liver and transported from the blood to the kidneys for excretion. Also, some is excreted by the gastrointestinal tract. Diseases such as hepatitis, cirrhosis of the liver and kidney problems cause an increase in urea in the blood
	Limitation	The minimum concentration of urea expected to move the motor is 50 mM, which is difficult and sometimes impossible to achieve in biological fluids, except in urine. High concentrations of urea increase the risk of meth-hemoglobin poisoning
	Mechanism	Urea is hydrolyzed to ammonium ions and bicarbonate anions by urease. By increasing the urease function, the formation of a local electric field due to the accumulation of ammonium ions is enhanced, which leads to the movement of the motor
Glucose	Properties	Blood glucose based on nutrients consumed and glucose production in the liver (through metabolic pathways such as glycogenesis, glycogenolysis and gluconeogenesis) is regulated. In solid tumors, glucose levels are lower than normal tissues due to a disordered vascular system, dysfunctional capillary substrate, and faster metabolism. Therefore, the tendency of glucose to enter tumors will be very high
	Limitation	Motors move (in response to the glucose gradient) toward areas with higher glucose concentrations. Therefore, the possibility of motor transmission to some cells or target tissues is difficult due to low glucose concentration. In addition, there is a possibility of hypoglycemia in the target tissue by the motors
	Mechanism	Glucose by GOx can be converted into glucuronic acid and H_2_O_2_. The generated H_2_O_2_ can subsequently be decomposed into harmless oxygen and water by CAT to power motors. In order to achieve a stable motion at a constant speed, the integration of GOx and CAT are considered
ATP	Properties	ATP is generated from ADP and mineral phosphate by F1F0-ATP synthase. ATP plays a key role in many processes such as muscle contraction, synthesis and degradation of biological molecules, and cellular signaling. Plasma ATP concentration in humans is determined at 1 mmol/L, while the intracellular concentration varies between 1–10 mmol/L based on cell function
	Limitation	An imbalance in the use of ATP increases the possibility of ADP accumulation, which inhibits adjacent bio-motors or motors. Also, adenosine accumulation due to off-targeted degradation of ATP outside the cell becomes highly toxic to cells
	Mechanism	ATP decomposition to release chemical energy
Acid	Properties	Biological acids such as stomach acid or acidic environments created in cancerous tissues or repairing tissues are a source of energy for nano-/micro-motors. The limitation of acidic environments in the body can provide the amount of nano-/micro-motors movement in a limited environment in addition to the possibility of targeting the nano-/micro-motors
	Limitation	An unusual corrosion of motors in an acidic environment can reduce their performance, and activated metal NPs activate unpredictable pathways. On the other hand, the reduction of the environment acidity caused by rapid evacuation of protons by motors has negative effects on the activity of some organs such as the stomach
	Mechanism	A reaction between the nano- or micro-motor and the surrounding protons, generates H_2_ bubbles

#### Magnetic-based propulsion

**Fig. 2:**
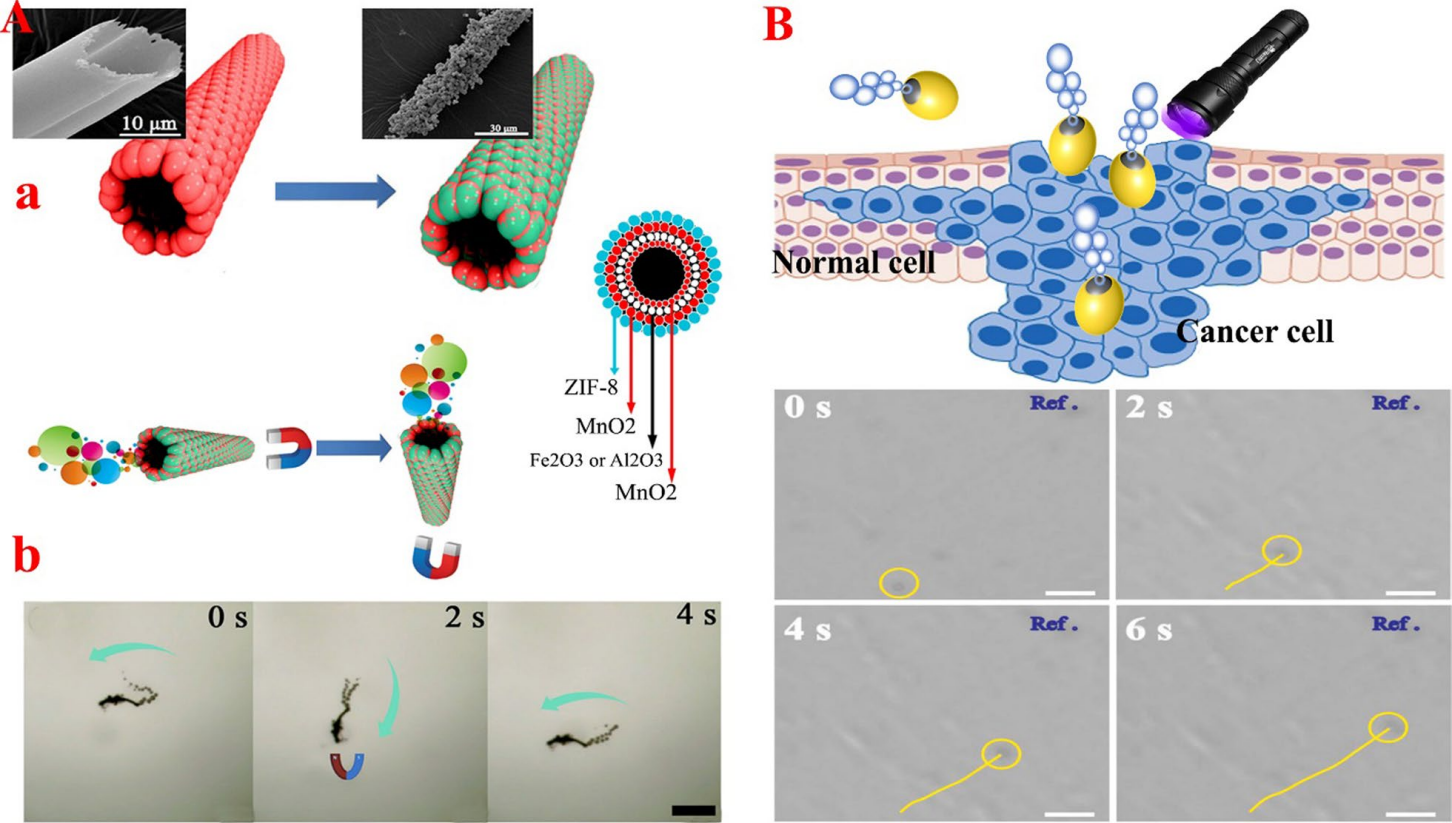
**A **MOF-NZs magnetic micromotors: a. Schematic illustration of the template-assisted synthesis method for the magnetic ZIF-micromotors and motion with direction control. b. corresponding time-lapse images of magnetic guided experiments in two seconds intervals. Figure adopted from Ref. [179]. **B **Janus micromotors: Schematic illustration of the light/gas cascade-propelled Janus micromotors to overcome sequential and trajectory pathway of Janus micromotors marked by yellow line under NIR irradiation (35.9 mW/cm^2^) along the time. Figure adopted from Ref. [221]

Catalytic NPs in nano-/micro-motors, face the challenge of short lifespan in the presence of environmental fuels. Therefore, the use of external factors such as magnetic field, which several studies show has no negative effect on biological activity [4, 180, 181], is could help to solve this problem. Also, due to the high flexibility and precise control of the magnetic field in the desired range, it is a suitable source for amplification and even independent movement of MOF-NZ nano-/micro-motors. By combining magnetic elements in motors and manipulating the external magnetic field, the direction and intensity of movement of MOF-NZ nano-/micro-motors in the target tissues can be adjusted optimally. In addition, the presence of magnetically sensitive metal NPs is expected to provide potential power for motors. The use of a magnetic field can effectively regulate the motion of MOF-NZ nano-/micro-motors uniformly in different physiological environments with varying viscosities. In this regard, Liu et al. [179] using γ-Fe_2_O_3_ in the framework of ZIF-8 were able to design MOF-NZ nano-/micro-motors based on the wet chemical method that has high controllability in the magnetic field to change the direction of motion (Fig. 2A). They also found that MOF-NZ nano-/micro-motors could be easily recovered by magnetic fields to prevent secondary contamination. On the other hand, their results showed that doxycycline loaded more on ZIF-8-based magnetic micromotors compared to γ-Fe_2_O_3_/γ-Al_2_O_3_/MnO_2_ microtubes due to the larger volume and surface area [179]. In this study, it was revealed that the use of H_2_O_2_ fuel propulsion, in addition to creating movement of the MOF-NZ nano-/micro-motors, allows more drug release. However, apart from the effect of H_2_O_2_ concentration on the speed of motors, no effect of magnetic field on the speed of MOF-NZ nano-/micro-motors was reported. However, the report shows that the speed of motors is almost two-fold than the speed of microtubes (150 µm/s vs. 72 µm/s) in a magnetic field [179]. Furthermore, using Fe_3_O_4_ NPs (for magnetic conduction) and Pt (for catalytic activity to produce propulsion) in the ZIF-8 framework, it was shown that Fe_3_O_4_-Pt-ZIF-8 rod micro-motors have the ability to rotate in the absence of H_2_O_2_ propulsion [182]. These micro-motors can follow a pre-programmed path. In this study, it was found that the use of a ZIF-8 framework increases the stability of Pt NZ in biological conditions, despite the challenge of Pt NPs in the acidic environment and the presence of H_2_O_2_ fuel [182].

Taken together, the above results show that the kinematic life of nano-/micro-motors is increased by synchronization of magnetic field and H_2_O_2_ decomposition. Increasing the lifespan of nano-/micro-motors can provide the ability to load more drugs into the target tissue, and provide better tissue access for surgeon motors, especially in more remote locations.

#### Light-based propulsion

The use of light is very important for driving nano-/micro-motors due to the simple regulation of intensity, frequency, polarization, and propagation direction (Fig. 2B). High biocompatibility properties along with reduced operating costs have led to the development and implementation of light radiation to move motors. The propulsion mechanisms of nano-/micro-motors based on light induction include electrophoretic, bubble, surface tension gradient, and deformation propulsions. In this regard, Troyano et al. [183] designed a light-sensitive MIL-88A@polyvinylidene difluoride that was able to change the angle of motion based on the swelling behavior caused by the re-configuration of the MIL-88A@polyvinylidene difluoride in the presence of UV rays and moisture. In this way, the MOF network structures were bent up to 90 degrees. In addition, the experiment revealed that increasing the humidity level from 60 to 90% increases the swelling behavior in MOFs, which indicates controllable behavior in humid environments. However, adjusting the MOFs motion by light can be challenged because of bending at the wrong time due to an increase in humidity (above 60%) in off-target locations. In the following, You et al. [184] using the dual function of GOx and CAT in the structure of MOF-NZ nanomotors with NaYF4:Yb,Tm@NaYF4 NPs /5,10,15,20-tetrakis(4aminophenyl)porphyrin@ZIF-8 were able to control the movement of nanomotors induced by 980 nm NIR light. They also found that the permeability of MOF-NZ nanomotors increases by up to 27% with light radiation. Nevertheless, light radiation is generally use for activities at the surface due to low penetration into the body. Therefore, the use of light in the transmission of MOF-NZ nano-/micro-motors will face challenges. However, the light-sensitive activity of MOFs in certain areas, such as the nucleus of skin cancer cells or tumor cells close to the surface, can lead to the elimination of a molecular abnormality. Because light-sensitive elastomers and polymers can undergo structural changes in the face of light, these can be used to change the direction of motion or the type of motion of MOF-NZ nano-/micro-motors [185, 186].

## Capabilities of MOF-NZ nano-/micro-motors in targeted treatment of cancers

The design and production of MOF-NZ nano-/micro-motors for operation in solvents and water is increasingly expanding. Despite the variety of nanomotor production methods, MOF-NZ nano-/micro-motors are currently being studied in three general formats, including self-propelled, synergism of self-propelled with external inductors, and nanorobots. However, the development of MOF-NZ nano-/micro-motors in therapeutic activities is greatly delayed. Therefore, much work is needed to expedite the arrival of nano-/micro-motors in therapeutic and diagnostic settings.

### Self-propelled MOF-NZ nano-/micro-motors

The use of the self-propelled method in MOF-NZ nano-/micro-motors is very important despite the challenges of therapeutic management such as direction of movement, permeability, and targeting, due to simple construction and no need for therapeutic accessories. In this line, Wang et al. [178] based on wet chemical method and ultrasonic system produced MOF-NZ micromotors in the form of ZIF-67 framework, which were not only capable of loading Fe oxide NPs and DOX with an efficiency of 40 to 43%, but also had high biocompatibility due to their degradability in the aqueous environment. They showed that due to the numerous cavities in the ZIF-67 framework, a high ability to transfer H_2_O_2_ into the ZIF-67/Fe3O4/DOX micromotors was provided, which ultimately resulted in long-term motion of up to 90 min with a speed of 15.3 to 76.3 μm/s (based on H_2_O_2_ concentration) [178]. The use of Fe NPs allowed a more accurate and powerful transfer of the MOF-NZ micromotors to the target cells via a magnetic field. In addition, their results recognized that drug release by two mechanisms included increasing the concentration and degradation of H_2_O_2_ in the cavities, and decomposition of MOFs by hydrolysis reactions in the framework [178]. Despite the lack of in vitro and in vivo experiments, ZIF-67/Fe_3_O_4_/DOX micromotors are likely to be useful in TDD, fluorescence imaging and MRI, induction of magnetic field to enhance motion, and because of the biocompatibility due to micromotors degradability, hope has been raised for the use in biomedical activities. In the next study, after examining the motor pattern of the CAT- poly(2-diisopropylamino)ethyl methacrylate@ZIF-L (CAT-PDPA@ZIF-L) micromotors by Guo et al. [187] they loaded the fluorouracil drug on the MOF-NZ micromotors to investigate the possibility of the transfer to cancer cells. Their results revealed that the motility of MOF-NZ micromotors increases with enhancing concentration of H_2_O_2_, and the rate of penetration of MOF-NZ micromotors into breast cancer cells cultured in a 3D chamber with a pH of 6.3 and a concentration of 150 µM H_2_O_2_ is boosted. Moreover, Guo et al. [187] examined the number of live/dead MCF-7 cells and found that MOF-NZ micromotors infiltrated breast cancer cells and delivered fluorouracil through micromotors breakdown, increasing cell death by 2 and 39% at pH of 6.3 and 7.4, respectively. However, the use of drug-free CAT-PDPA@ZIF-L micromotors had no significant effect on MCF-7 cell death. Therefore, their results indicate the effect of environmental acidity on the movement of MOF-NZ micromotors; with increased acidity death rate of breast cancer cells is significantly reduced. Also, these findings indicate the accuracy of the MOF-NZ micromotors in loading of anticancer drugs in cancer cells with an acidity between 6 and 6.5 [187]. Likewise, Gao et al. [176] using CAT and succinylated β-lactoglobulin loaded on the ZIF-L framework, were able to develop CAT-β@ZIF-L micromotors sensitive to pH and H_2_O_2_ concentration, which in addition to sensitivity to very low toxicity, have promising chemical and thermal stability. The dual reaction of the synthesized MOF-NZ micromotors to pH and H_2_O_2_ for the programmed degradation of the motors results in accurate drug loading in the target cells. Furthermore, it was shown that the ZIF-L has no biocatalytic activity and that the catalytic activity was performed only by CAT [176]. Thus, structural modification of succinylated β-lactoglobulin as a biological gateway with change of pH can cause MOF-NZ micromotors to turn off or turn on based on H_2_O_2_ access. After loading DOX in CAT-β@ZIF-L micromotors, Gao et al. [176] showed that the distribution profile of DOX in Hela cells is two-stage and effectively reduces the survival of cancer cells. In the first stage, the drug is partially released from MOF-NZ micromotors due to the presence of peroxides. Then, in the second stage, DOX is widely released by long-term retention of CAT-β@ZIF-L micromotors in HeLa cells causing cell death.

### Synergism of self-propelled with external inductors

**Fig. 3:**
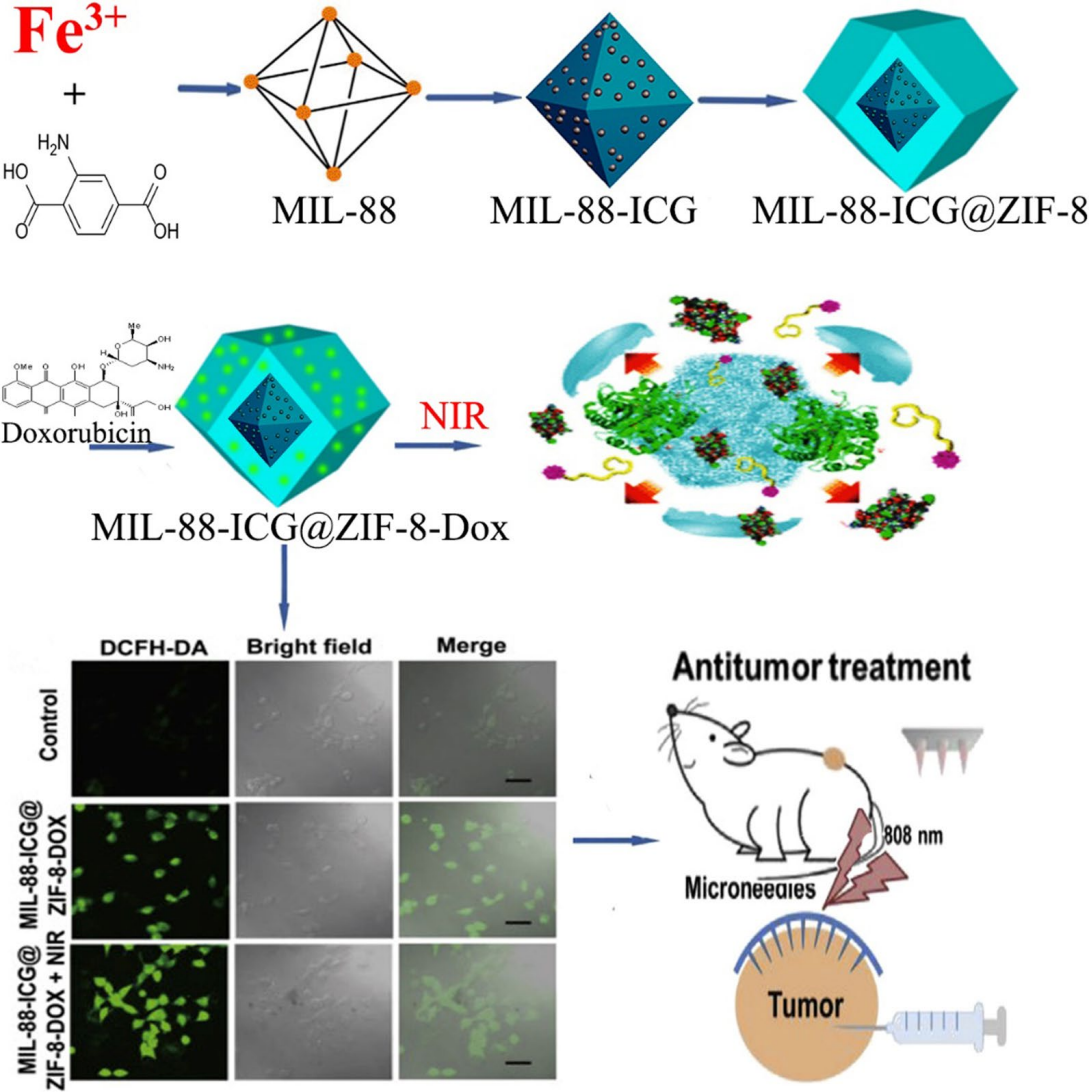
Schematic illustration of the synthesis process of MIL-88-ICG@ZIF-8-DOX, the speculation about the drug release mechanisms of MIL-88-ICG@ZIF-8-DOX and in vivo antitumor experimental by microneedles containing motors after determining 4T1 cell viability with treatments. Figure adapted with permission from Ref. [188]

In order to enhance the penetration and effectiveness of nanomotors along with motion control, the use of external inductors is very exciting. In this regard, based on the one-pot method, You et al. [184] generated MOF-NZ nanomotors based on the ZIF-8 platform containing CAT, GOx, 5,10,15,20-tetrakis(4-aminophenyl)porphyrin (TAPP, a typical photosensitizer) and NaYF4:Yb,Tm NPs (UCNPs: thermally synthesized with hexagonal morphology) which in addition to optimal chemical and thermal stability have high biocompatibility due to very low toxicity. Their results also show that the motion of the nanomotors is dependent on the increase in H_2_O_2_ concentration, but their direction of motion was more random and based on the asymmetric distribution of CAT on the UCNPs/TAPP@ZIF-8@CAT/GOx nanomotors. You et al. [184] explained that due to the accumulation of fluorescence light emitted by TAPP in cancer cells, MOF-NZ nanomotors have significantly entered the cytoplasm of Hela cells. Besides, their report indicates a close relationship between GOx activity in the production of H_2_O_2_ and the degradation by CAT for nanomotor motion. On the other hand, GOx activity increases starvation in Hela cancer cells by consuming glucose substrate. Furthermore, the results indicate a significant increase in nanomotor toxicity in cancer cells with NIR radiation, which reduced the survival rate of HeLa cancer cells below 79%. Ultimately, their report shows that by combining the activities of CAT, GOx, and NIR irradiation, the survival rate of cancerous cells decreases below 25%, which significantly extends the therapeutic effect in uterine cancer by MOF-NZ nanomotors [184]. Decreased cell viability by UCNPs/TAPP@ZIF-8@CAT/GOx nanomotors due to synchronization of therapeutic activities is associated with increased oxygen concentration by H_2_O_2_ decomposition and helping to increase the cellular uptake of nanomotors to produce cytotoxic molecules (single oxygen) via NIR irradiation. Recently, using the MIL-88 and ZIF-8 frameworks, Wu et al. [188] designed a MOF-NZ nanomotors with two cores MIL-88 as the inner part and ZIF-8 as the outer shell, which in addition to TDD, enables combination of PTT/PDT with chemotherapy for the treatment of breast cancer (Fig. 3). In the MOF-NZ nanomotors, Indocyanine green (ICG) in the inner core (with 88.96% loading efficiency) during PDT produces ROS agents and the outer core containing DOX (with 87.84% loading efficiency) induces apoptotic cell death. Their findings show that MIL-88-ICG@ZIF-8-DOX nanomotors significantly increase the release rate of DOX from 25% at a pH of 7.4 to 60% at a pH of 5 due to breaking of bonds between DOX and Zn ions [188]. Moreover, it was found that NIR radiation increases the rate and speed of drug release from the MOF-NZ nanomotors. In addition, Wu et al. [188] demonstrated that the oxygen required for the production of ROS as well as the formation of bubbles to move the MOF-NZ nanomotors is provided by NIR irradiation of MIL-88. In vitro studies demonstrated that MOF-NZ nanomotors effectively penetrated into 4T1 cancer cells by releasing ICG through PTT, then by releasing DOX, increasingly enhanced apoptotic cell death in 4T1 cancer cells [188]. However, the results show that MIL-88-@ZIF-8 nanomotors without drug and NIR irradiation had no significant effect on 4T1 cells. Furthermore, in a mouse model, Wu et al. [188] explained that the antitumor effect of MOF-NZ nanomotors combined with NIR irradiation was significantly prominent compared to the control groups, free drug, nanomotors without DOX, and NIR irradiation alone. Nevertheless, their results show that the nanomotors administration method is very effective in inhibiting tumor growth. The administration of MOF-NZ nanomotors via microneedle effectively reduced breast cancer growth by 82.1, 20.4 and 41.2% compared respectively to the control group, the injectable and intramural methods [188].

### MOF-NZ microrobots

Because microrobots based on MOF-NZs are a good candidate in TDD and molecular surgery due to the controllable movement of microrobots, especially in long-term activities, the use has recently received considerable attention. In this line, based on the two-photon polymerization stereolithography method, a 3D structure with ZIF-8 crystals containing Ni and titanium and spiral swimmers (artificial bacterial flagella (ABF)) was designed as a ZIF-8@ABF microrobots that is biocompatible, sensitive to environmental pH and stable against thermal and chemical stresses [189]. In this experiment, Wang et al. [189] showed that the ZIF-8@ABF microrobots have three types of motion, including wobbling (below 10 Hz magnetic field), corkscrew (between 10 and 40 Hz magnetic field) and step-out mode (above 40 Hz magnetic field). With an increase in magnetic field, the speed of ZIF-8@ABF microrobots movement increased significantly, and by changing the direction and frequency of rotation of the magnetic field, the movement of microrobots could be precisely controlled. On the other hand, their results demonstrated the transfer of Rhodamine B (RhB) dye to breast cancer cells (MDA-MB231) cultured in petri dishes by moving the RhB@ZIF-8@ABFs microrobots directly to the target cells without entering adjacent cells [189]. With increasing acidity of the environment, the movement of the RhB@ZIF-8@ABFs microrobots towards the target cells became faster, and at pH 6, the highest drug loading was observed in cancer cells. While, micromotors remained intact at neutral acidity (pH 7.4). Finally, Wang et al. [189], by applying microfluidic kits, revealed that the designed RhB@ZIF-8@ABFs micromotors can selectively pass through microfluidic channels. This study can be a potential model for targeting the movement of MOF-NZ micromotors in medical settings. Similarly, Terzopoulou et al. [190] designed a nanorobots (with dimension of 150 nm) by loading Fe, DOX, and gelatin methacryloyl in a ZIF-8 framework based on the two-photon polymerization stereolithography way, which is not only biodegradable and biocompatible, but also effectively increases drug loading in MDA-MB231 breast cancer cells through enzymatic degradation of DOX@Fe@ZIF-8@GelMA nanorobots in acidic media. Increasing the acidity of the environment, especially at an acidity of 5 to 6, the drug is effectively released from the MOFs nanorobots. In addition, their results show a reduction in MDA-MB231 cancer cell survival by up to 65% compared to controls, whereas DOX-free nanorobots reduced cell survival by only 10% [190]. Also, in this study, it was revealed that the motion of the DOX@Fe@ZIF-8@GelMA nanorobots can be precisely controlled by the magnetic field, which can be obtained by the three mechanisms described above [190].

## Biological barriers in the transfer of nano-/micro-motors

Although in the last eight years, MOF-NZ nano-/micro-motors have received much attention due to the high cavities for drug loading, and degradation under physiological conditions, intelligent mobility and easier production, activity in this field has been delayed due to inability to pass the blood–brain barrier, immune system clearance, biological deposition, barriers of vascular systems and cellular membranes, blood flow, and transfer to similar tissues (resulted from similarities in the acidity of the environment or biological molecules). Therefore, in order to use the nano-/micro-motors for medical indications, it is necessary to remove all biological barriers and focus on applied studies in a repetitive manner.

**Fig. 4:**
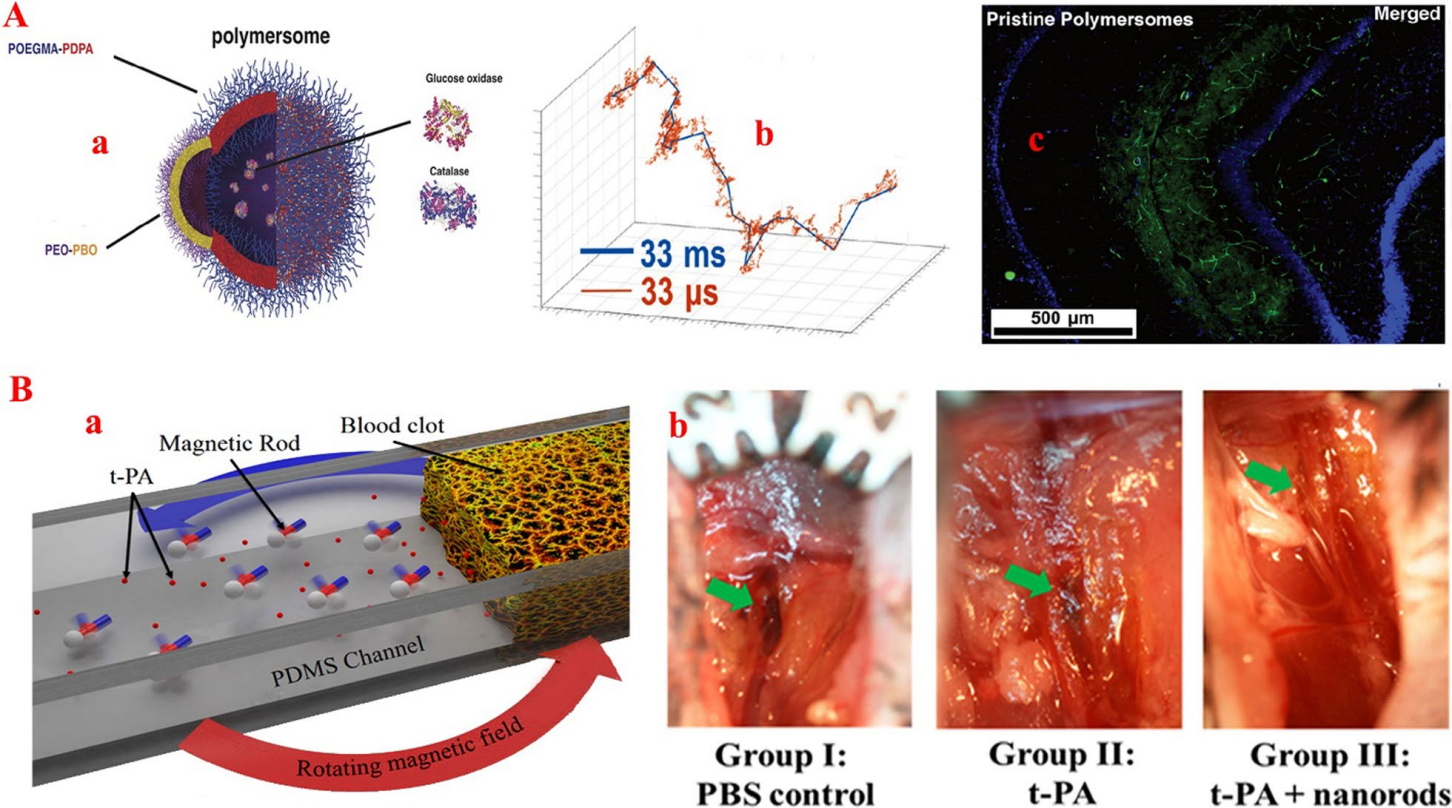
**A** Schematic representation of a chemotactic polymersome using a combination of membrane topology formed by PEO-PBO copolymers mixed with POEGMA-PDPA copolymers. The polymersomes encapsulate glucose oxidase and catalase enzymes, b. A single simulated 3D trajectory shown with temporal steps of 33 ms (blue line) and 33 ms (orange line), and c. Immunofluorescence histologies of rat hippocampus sections treated with pristine asymmetric POEGMA-PDPA/PEO-PBO polymersomes loaded with GOx and CAT (figure Adopted from Ref. [192]. **B** a. schematic view of nanomotor enhanced thrombolysis in fluidic channels. b. Thrombolysis evaluation in the right femoral vessels of mice belonging to three groups associated with different administrations. The green arrows indicate the inducted region in the femoral vessels of C57/BL6 mice. Reprinted with permission from Ref. [202], Copyright (2019) Elsevier B.V)

The most difficult barrier for the delivery of a drug to the target tissue is the blood–brain barrier, which includes endothelial cells, astrocytes, and pericytes in the vascular wall. This physiological barrier selectively facilitates and regulates the transport of various molecules that are less than 500 Da [191]. Therefore, the treatment efficacy of brain tumors with chemotherapeutics that are more than 500 Da will be generally low. Despite the fact that nanotechnology has been pursuing TDD through this physiological barrier for many years, this approach still faces the problem of low drug delivery efficiency and poor targeting. For this purpose, the use of the glucose concentration gradient is a recommended solution for TDD through the blood–brain barrier. In this regard, Joseph et al. [192] developed a hybrid micromotor based on the loading of PMPC-*b*-PDPA/PEO-PBO copolymers and DNA with GOx/CAT on the polymersome, which move in response to an endogenous glucose gradient to places with higher glucose concentrations (Fig. 4A). This process caused the designed hybrid micromotors to penetrate four times as much into brain tissue compared to other NPs or motors due to the high accumulation of glucose [192]. When the nano-/micro-motors entered the cerebral vessels, the movement of the motors into the brain increased due to the increase in the concentration gradient of glucose from the center of the vessel to the membrane and into the tissue. This report indicates an increase in the penetration rate into the blood–brain barrier in the presence of GOx, which can be considered a potential solution for MOF-NZ nano-/micro-motors.

The body's second major barrier inhibiting TDD to tissues is clearance through the immune system. It has already been shown that chemical modification or the use of polymer coatings on particles can prolong presence in the bloodstream and impairs interaction with phagocytes [193, 194]. With this strategy, MOF-NZ nanomotors can be effectively rendered hydrophilic and provide a so-called stealth appearance rendering nanomotors invisible from the immune system. In addition, the formation of a corona, which is the adherence of blood components such as proteins, to the outside of injected NPs alters or impairs the performance of NPs, and also nanomotors, which can be solved by using natural cell membranes to coat the NPs [195]. For example, Shao et al. [196] and Xuan et al. [197] used red blood cells membranes to coat the structure of Janus capsule micromotors, not only increasing the biocompatibility of micromotors and their stability in the blood, but also significantly reducing bio-fouling and occurrence of thrombosis.

In addition to immune clearance and corona proteins, MOF-NZ nano-/micro-motors must overcome the anatomical structure of blood vessels and the fluid properties of blood to reach target tissues. Barriers of vascular and blood systems such as the diameter of some capillaries which in some cases are a few micrometers and the velocity of blood that in most cases is higher than the velocity of motors, can cause serious problems in controlling MOF-NZ nano-/micro-motors movements. Failure to pay attention to the above problems can lead to embolic complications and stroke. One of the recommended ways is to use bio-hybrid nano-/micro-motors. The use of normal blood cells (e.g., neutrophils), DNA, and exosomes due to their natural navigability, can after entering the bloodstream and activated by cytokines, deliver the MOF-NZ nano-/micro-motors to tumor tissues [198–200]. However, increasing the size of the carriers and changing the type of nano-/ micro-motor movement caused by the absorption of blood components to these motors is a major obstacle for this approach. For instance, Venugopalan et al. [201] determined that the change in micromotors motion to stick–slip dynamics, which is not observed in the case of simple Newtonian fluids, is related to the colloidal occlusion of blood cells in the micromotors. Another method is to use external inductors to change the direction and enhance the movement of the MOF-NZ nano-/micro-motors, even against vascular currents. For example, Cheng et al. [202] developed nanomotors that were transported through the bloodstream to target blood clots (Fig. 4B). They then accumulated these motors around the blood clots by applying a magnetic field and accelerated thrombolysis.

One of the most common biological barriers in the body is the bilayer cell membrane, which relatively and actively prevents the entry of some compounds. Nowadays, most therapeutic drugs enter the cytosol and nucleus continuously through nanotechnology and the induction of external factors such as PDT/PTT and magnetic fields [19, 203, 204]. Merging with cell membranes is the simplest way to transfer drug or chemical compounds. However, in the field of micromotors, the use of increasing the concentration of single oxygen is a suitable solution to increase the penetration of the MOF-NZ nano-/micro-motors into the cell, which is provided by the activity of CAT and external inductions including NIR/UV irradiation. In this regard, Guo et al. [187] and You et al. [184] drastically increased the entry of the MOF-NZ nano-/micro-motors into the cell by increasing the single oxygen induced by the simultaneous activity of CAT and NIR irradiation. Furthermore, the use of peptide coating and modification of the nano-/micro-motors surface with ligands can enhance the intracellular diffusion coefficient [193]. However, it has been observed that in some cases peptides do not effectively promote targeted penetration and may even enhance immune clearance [205].

## Challenging and opportunities

Overall, incredible advances have been made in the field of micromotors, especially in the field of pollutant removal, but the development of MOF-NZ nano-/micro-motors is still in its infancy and is much more limited in the field of biomedical activities. Therefore, much research is needed to explain challenges such as (1) the availability of MOFs for specific purposes, (2) the reversible and irreversible changes of MOFs enzymatic sites in physiological environments, (3) mechanical studies to explain the power of MOF-NZ nano-/micro-motors, (4) controlling and fine-tuning pore structures, (5) explaining the synergistic effects of components in the frameworks on motor performance, (6) facilitating the production of MOF-NZ nano-/micro-motors, (7) improving drug loading levels, (8) increasing the long-term motion of MOF-NZ nano-/micro-motors, and (9) integration of TDD and imaging systems in MOF-NZ nano-/micro-motors. However, there are three critical challenges to MOF-NZ nano-/micro-motors therapeutic activities.

**Fig. 5:**
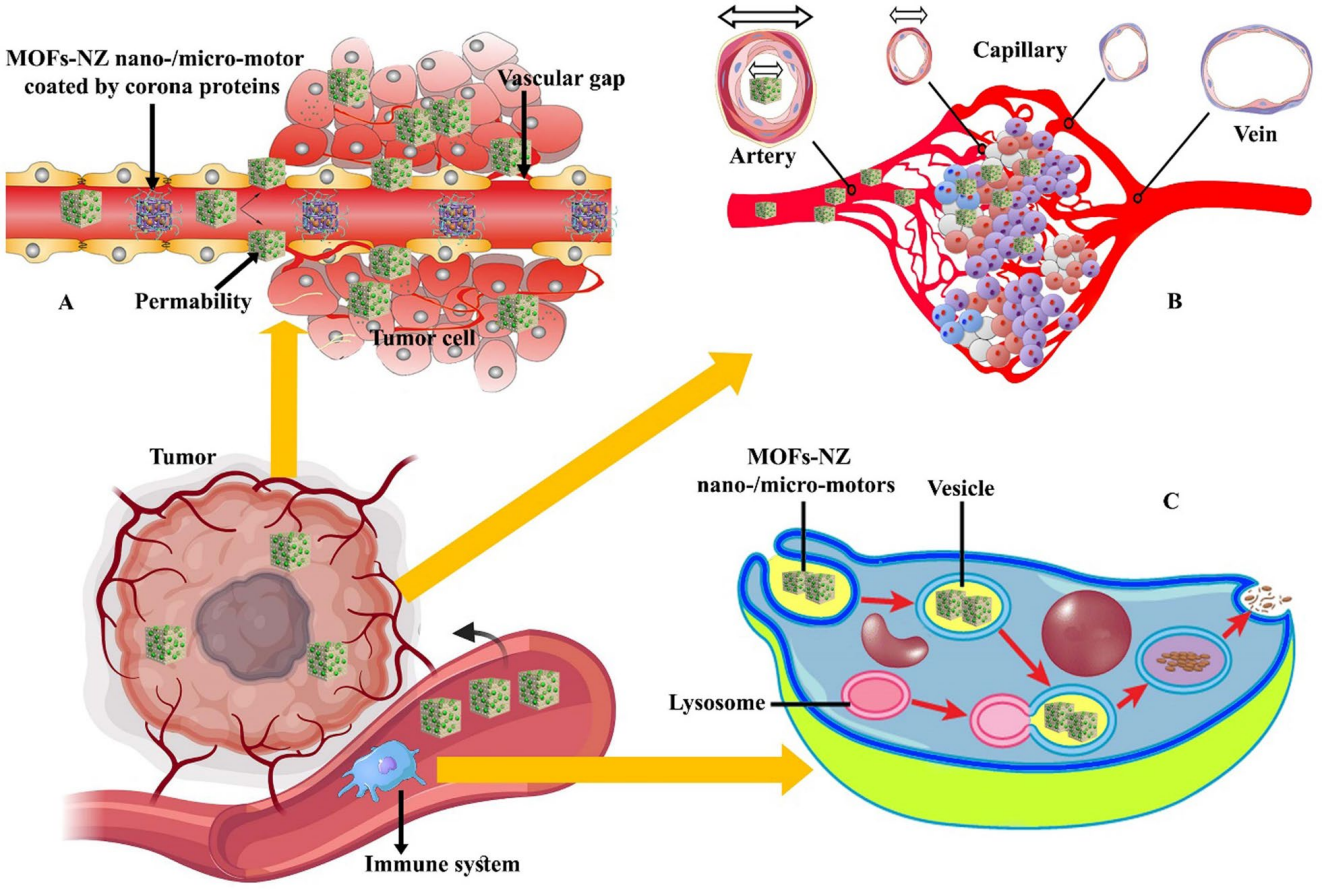
Schematic representation of common challenges in transporting MOF-NZ nano-/micro-motors in cancerous tissue. **A** The presence of corona proteins on the motors reduces the possibility of penetration into tumor tissue. **B** Increasing the size of the motors reduces access to the depths of cancerous tissue due to the reduction in capillary thickness and **C** One way to eliminate the motors is to use the immune system to digest them in the digestive vesicles and convert into non-toxic elements

It can be said that the first condition for the treatment of cancer is the potential targeting of nano-/micro-motors to the tumor mass with high therapeutic ability and stability in the body. The stability of the MOF-NZ nano-/micro-motors in the body is a prerequisite, which is challenged according to Sect. 5, including phagocytosis by the immune system, inactivation of particles by corona proteins, and clearance through organs such as kidneys and liver. Among biological barriers, corona proteins, due to the deposition of these protein on the surface of MOF-NZ nano-/micro-motors and the reduction of chemical reactions can simply reduce the inherent activity of the motors (Fig. 5A). On the other hand, the removal of MOF-NZ nano-/micro-motors by the immune system based on the breakdown of nanomotors in the acidity of lysosomes is another serious challenge that can limit the nanomotors' access to tumor tissues. Reports indicate that the immune system removes over 95% of the NPs used, which is affected by nature, size and performance of the motors [206, 207]. However, the reported papers do not address these two major challenges. Although various reports indicate that the use of biocompatible and biodegradable materials in the framework of MOFs and their surface modification with coatings can significantly reduce these problems, no report of this procedure has been published for the construction of MOF-NZ nano-/micro-motors [208–211]. Therefore, most of the capabilities of micromotors that are reported in the laboratory are not suitable in vivo because of above mentioned challenges.

The second major challenge of using MOF-NZ nano-/micro-motors is to perform therapeutic activities according to predetermined schedules, such as motors entering the appropriate tumor site at the appointed time, drug release with a defined mechanism and time, and performing concurrent therapeutic activities. Because blood vessels in tumor tissues lack the necessary integrity (Fig. 5A) [212], it is thought that motors can easily enter the tumor mass. But motor size similar to NPs [213] can be a major challenge to penetrate the MOF-NZ nano-/micro-motors deep into the tumor tissue in the early stages of formation (Fig. 5B). Lack of drug release deep into the tumor tissue increases the likelihood of tumor recurrence. For this purpose, the use of MOF-NZ nano-/micro-motors containing several powerful propellants, and along with induction of motion by external inductors this can eliminate the problem. However, there is still no promising report on successful propulsion in solid tumor models.

The next challenge is the release of drugs by MOF-NZ nano-/micro-motors in solid tumors, which is based on changing the physical structure of motors via changing the environment pH and temperature. Although reports from in vitro model indicate that drug release is performed effectively [188, 190], the drug release profile in vivo and the possible lack of drug release due to double chemical bonding have not been reported. The use of external inducers such as irradiation, magnetic field and ultrasound can effectively increase the level of drug release [214, 215]. However, the use of external inducers requires penetration into solid tissue as well as motor stability in in this tissue. The use of inductors increases the possibility of increasing the movement of nano-/micro-motors to other centers and their augmented accumulation at the induction site due to the activation of propellants. On the other hand, the likelihood of unplanned chemical behaviors or interactions due to inductor fluctuations can generate some concerns. With the few papers reported, it is not possible to provide a definitive answer on the mentioned challenges.

One of the next important challenges in using a MOF-NZs nano-/micro-motor is the end of therapeutic operations and the removal of the motor by simple methods without toxicity. But, in the production of nano-/micro-motors, the use of metal NPs for catalytic activities, improving the strength and ability to form chemical bonds is common and necessary. For instance, the use of metal NPs such as Mg and Al to form bubbles [216], metal oxides such as TiO_2_ and MnO_2_ for catalytic activity [217], and the use of magnetic particles such as Fe for the simultaneous activity of external magnetic inducers [218] are considered. Since larger NPs can enhance their activity as propellants [219], the challenge of their presence will remain after MOF-NZs nano-/micro-motor degradability. Some people believe that motors can be recovered after the end of therapeutic activities [220], but it is technically challenging due to the location, time and method of collection. Also, relying on the immune system to remove the MOF-NZs nano-/micro-motor is an approach (Fig. 5C) that contrasts with hiding the motor from the immune system in the early stages. However, biodegradable polymers can be used as a coating that separates from the MOF-NZ nano-/micro-motors after the motor stops working. Then, the nano-/micro-motors are removed as a residual unit by the immune system. Overall, reducing the shelf life of motors after therapeutic application is a serious challenge due to their mobility and the possibility of performing catalytic activities at inappropriate locations.

In order to overcome the mentioned challenges, it is necessary to provide multi-purpose MOF-NZ nano-/micro-motors with the capability of controllable and automatic movements, biosensing, TDD and even imaging, which is obtained from the integration of different scientific fields. Therefore, extensive efforts and new innovations are needed to use MOF-NZ nano-/micro-motors in order to be used clinically. However, experiments have shown that there is promise at the horizon for the use of MOF-NZ nano-/micro-motors, especially in TDD and even disease diagnosis.

## Conclusion

In this review, the function of MOF in the treatment of cancers, mechanism of action, and its manufacturing methods were briefly considered. Because reported papers show that MOF-NZs are highly potent in the treatment of cancers through TDD and catalytic activity, their use as nano-/micro-motors can result in development of potential platforms for the treatment of several types of cancers. MOF-NZ nano-/micro-motors with the ability to move automatically through propulsion can easily interact with different parts of the body according to predetermined programs and provide therapeutic functions. However, in this review, by highlighting the various challenges posed by biological barriers such as blood–brain barrier, cellular barriers, vascular structure and blood flow, and immune system clearance, we conclude that effective treatment by MOF-NZ nano-/micro-motors requires precise control of all aspects. Also, important challenges such as location, the ability to execute commands, and control over ending of role and presence of MOF-NZ nano-/micro-motors have limited the possibility of their extensive activity in vivo. In this review, we reveal that the use of external inductors along with the use of biodegradable structures can provide some of the answers. In addition, the reports presented in the discussion show that facilitating the movement of motors based on the simultaneous loading of enzymes and NPs with catalytic properties can intensify the processes of TDD and release in target tissues and solve the existing challenges. However, understanding the precise operation of MOF-NZ nano-/micro-motors in the body requires extensive research both in vitro and in vivo. Although there have been various reports on the use of motors in vitro, there is a long way before effective application in vivo. Overall, this study reports on the limitations and benefits of MOF-NZ nano-/micro-motors compared to passive MOFs, and may encourage researchers to apply MOF-NZ motors for the treatment of cancer.

## Data Availability

Not applicable.

## Abbreviations

ADP/ATP: Adenosine di/tri phosphate
Au: Gold
Al: Aluminum
Apt: Aptamer
Ag: Silver
BPQDs: Black phosphorous quantum dots
BQ: Black phosphorus Quantum dot
BSA: Bovine Serum Albumin
BM: BSA-stabilized MnO_2_
CNT: Carbon nanotube
CAT: Catalase
Cs: Cerium
CDT: Chemodynamic therapy
CHT: Chemotherapy
Co: Cobalt
CT: Computed tomography
CuO: Copper oxide
CpG: Cytosine-phosphate-guanine sequence
DSP: Diamminedichlorodisuccinato-Pt
DHA: Dihydroartemisinin
DOX: Doxorubicin
Fc: Ferrocene
Fe: Iron
FA: Folic acid
Gd: Gadolinium
GOx: Glucose oxidase
GSH: Glutathione
GNR: Gold nanorod
GSNO: S-Nitrosoglutathione
Hb: Hemoglobin
HRP: Horseradish peroxidase
HA: Hyaluronic acid
H_2_O_2_: Hydrogen peroxide
IMT: Immunotherapy
ICG: Indocyanine green
IL: Ionic liquid
Ir: Iridium
MRI: Magnetic resonance imaging
Mn: Manganese
MIL: Materials Institute Lavoisier
MS: Mesoporous Silica
MOF-NZs: Metal organic framework-nanozymes
MPN: Metal polyphenol network
MB: Methylene blue
MDT: Microwave dynamic therapy
NPs: Nanoparticles
NCPs: Nanoscale coordination polymers
NIR: Near infrared
Ni: Nickel
Pd: Palladium
PDT: Photodynamic therapy
PTT: Photothermal therapy
Pt: Platinum
PAH: Poly(allylamine hydrochloride)
PVP: Poly(vinyl pyrrolidone)
PDA: Polydopamine
PEG: Polyethylene glycol
PLA: Polylactic acid
PCN: Porphyrin metal–organic frameworks
PET: Positron emission tomography
PB: Prussian blue
RT: Radiotherapy
ROS: Reactive oxygen specious
Si: Silicon
SDT: Sonodynamic therapy
SOD: Superoxide dismutase
SERS: Surface-enhanced raman spectroscopy
TDDSs: Targeted drug delivery systems
TPP: Tetraphenylporphyrin
TPZ: Tirapazamine
TB: Toluidine blue
TCPP: Tris(2-chloroisopropyl)phosphate
UV: Ultra violet
US: Ultrasound
ZIF: Zeolitic imidazolate framework
Zr: Zirconium
